# A Results-Oriented Approach for the Animal Welfare Measure of the European Union’s Rural Development Programme

**DOI:** 10.3390/ani11061570

**Published:** 2021-05-27

**Authors:** Angela Bergschmidt, Solveig March, Kathrin Wagner, Jan Brinkmann

**Affiliations:** 1 Thünen Institute of Farm Economics, Bundesallee 63, 38116 Braunschweig, Germany; 2 Thünen Institute of Organic Farming, Trenthorst 32, 23847 Westerau, Germany; solveig.march@thuenen.de (S.M.); kathrin.wagner@googlemail.com (K.W.); jan.brinkmann@thuenen.de (J.B.)

**Keywords:** animal welfare, Rural Development Programme (RDP), action-oriented support, results-oriented support, dairy cows, Welfare Quality^®^, welfare indicators

## Abstract

**Simple Summary:**

So far, the animal welfare support measures of the EU’s Common Agricultural Policy for dairy cows have been action-oriented. Farmers receive a payment for their welfare friendly housing system or management (inputs). As the actual animal welfare output is not considered, these support-measures can create good conditions for animal behaviour, but are not able to address animal health. This would be possible in a results-oriented support measure, where the payment is linked to the output (animal-based indicators). With the aim of making animal welfare support more effective, we therefore examined which indicators would be suitable for a results-oriented support measure and how such a measure would have to be designed to encompass all dimensions of animal welfare: animal health, behaviour and emotional state. In a multi-stage selection process involving scientists and practitioners, 10 indicators were identified as appropriate. Because these only cover animal health, a combined action- and results-oriented measure is recommended, in which the dimensions “behaviour” and “emotional state” are addressed via action-oriented requirements and the dimension “health” via results-oriented indicators. With the results of our research, we provide the knowledge base for policy makers and administrators to implement agricultural support policies which can effectively improve the welfare of dairy cows.

**Abstract:**

Farm animal welfare is a major concern to the European Union’s citizens, addressed in the Rural Development Programmes by a specific animal welfare support measure. Previous evaluation results reveal that the implemented action-oriented measures fail when it comes to improving animal health, an important dimension of animal welfare. Results-oriented measures could compensate for this deficiency, but little is known about their design. In order to improve the effectiveness of current animal welfare measures for dairy cows, we analysed the elements of such a measure in an interdisciplinary, application-oriented research project involving agricultural economists and livestock scientists. We have used a mixed methods approach including a written Delphi survey, group-discussions and on-farm data-collection to select suitable indicators, develop an approach for the identification of threshold values and to design a support measure. Results suggest that, in animal welfare support measures, action- as well as results-oriented elements are necessary to address all dimensions of animal welfare.

## 1. Introduction

European citizens are concerned about farm animal welfare. According to a recent Eurobarometer survey, 82% of the respondents’ state that “in general, the welfare of farmed animals should be better protected than it is now” [1] (p. 12). Scientific studies have detected a large number of animal welfare problems on European farms. These range from widespread incidences of disease, such as mastitis in dairy cows; high prevalence of injuries caused by inadequate housing conditions, such as foot pad lesions in poultry; and behavioural disorders, such as tail biting in pigs or feather pecking in laying hens; to routinely conducted mutilations such as castration, dehorning and tail docking [2,3,4]. Animal welfare has a number of attributes which characterise it as a public good in short supply [5,6,7], thus calling for policy intervention to ensure adequate provision. Various instruments can be implemented to support this aim. In addition to a tightening of animal welfare legislation and the provision of appropriate information for consumers (i.e., animal welfare labelling), these include support measures for farmers. Such measures have the advantage of compensating for the higher costs of welfare-friendly husbandry without challenging the competitiveness of animal production on international markets.

The EU’s Common Agricultural Policy (CAP) makes up for over one third of the EU’s budget, its two pillars, the Direct Payments and the Rural Development Programmes (RPD) channelling roughly 60 billion Euro of support for farmers each year [8].

**Figure A1:**
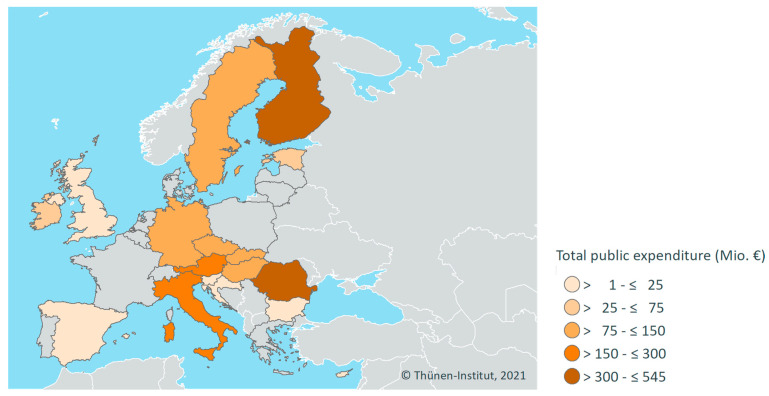
Total public expenditure for Measure M14—Animal Welfare in the EU Member states in 2014–2019; Source: https://cohesiondata.ec.europa.eu/ (accessed on 19 January 2021). Data and Maps, esri (2021).

**Figure A2:**
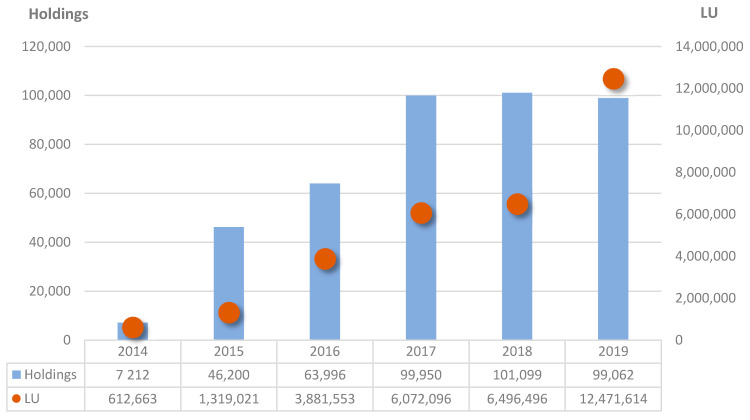
Number of supported Holdings and supported Livestock Units (LU) for Measure—Animal Welfare in the EU Member states in 2014–2019.

In the RDP, one specific measure, “Animal welfare” (M14, Article 33 in [9]), has been programmed to improve animal welfare. Other RDP-measures which can be implemented to improve farm animal welfare are farm investment support, knowledge transfer and advisory services. However, these are predominantly used to increase the short-term competitiveness of the supported farms. With the measure M14, farmers “going beyond the relevant mandatory standards” can receive annual payments per livestock unit (LU). So far this measure only accounts for a small percentage of the RDP budget and is not implemented in all member states [10], yet it has gained importance in recent years, with the number of implementing countries, supported holdings, and Livestock Units, as well as public expenditure, increasing steadily (see Figure A1 and Figure A2 in Appendix A for an overview).

In future, this measure or alternative animal welfare support such as the ones planned in the framework of the Eco-Schemes [11] could become even more important as animal welfare remains an issue of high public interest and the existing problems persist. As with all RDP measures, the definition and implementation of the support takes place on the level of the EU-Member State or even at the regional level (i.e., in Germany, the federal states; in Italy, the regions). Up to now, measure M14 is almost exclusively implemented as an action-oriented support-measure and is targeted towards dairy cows in most countries.

Action-oriented measures are the common policy approach for the provision of public goods in the EU’s RDP and are widely used in agri-environment schemes. They compensate farmers for the higher cost incurred dependent on the fulfilment of specific conditions (inputs). In contrast, action-oriented measures pay for the achievement of a specific output, in the biodiversity context, for instance, for the occurrence of endangered flora in a habitat.

Participating farms commit themselves to requirements such as access to pasture or the provision of straw bedding (for an example of the measure’s requirements, see Appendix A). The evaluation of M14 in Germany has shown that this action-oriented approach can provide good conditions for animal behaviour, but is not able to address animal health [12]. Results-oriented measures could compensate for this deficiency, as animal health can be measured adequately using animal-based indicators. The inclusion of results-oriented elements could therefore make M14 more effective, “ensuring that farmers are paid for provision rather than for performing management behaviours that may, or may not, lead to provision” [13]. Results-oriented approaches are widely used in animal welfare research (“animal-based indicators”) but are novel with respect to animal welfare support measures.

With the aim of providing a knowledgebase for the improvement of the animal welfare effects of agricultural policy support measures, this paper presents the results of an interdisciplinary, application-oriented research project involving agricultural economists and livestock scientists. The necessary elements of a results-oriented animal welfare measure for dairy cows have been analysed and recommendations formulated to increase the effectiveness of the current RDP’s animal welfare support.

The project was divided into two phases, (1) the identification of suitable indicators and (2) the determination of threshold values and measure design.

### 1.1. Identification of Suitable Indicators

Different concepts exist to operationalise animal welfare. The most widely used are the “five freedoms” [14] and Fraser’s [15] multi-dimensional model.

**Figure 1:**
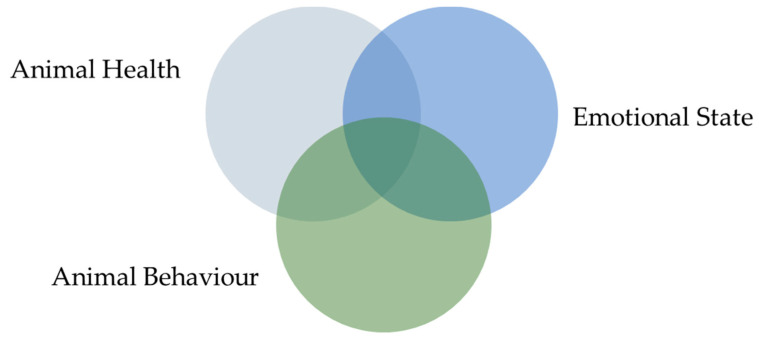
Fraser’s multi-dimensional animal welfare model ([15] modified).

Both concepts address the same animal welfare issues, but Fraser’s approach makes the missing possibility of compensation between the different animal welfare dimensions obvious. Therefore, a good status of animal health and animal behaviour and emotional state is necessary in order to accomplish high animal welfare (the intersection of the circles in Figure 1).

Based on these concepts, the World Organisation for Animal Health (OIE) has drawn up the following definition: “Animal welfare means the physical and mental state of an animal in relation to the conditions in which it lives and dies. An animal experiences good welfare if the animal is healthy, comfortable, well nourished, safe, is not suffering from unpleasant states such as pain, fear and distress, and is able to express behaviours that are important for its physical and mental state.” [16]. Common to all three definitions and of crucial importance to our research is the multidimensional nature of animal welfare.

Animal welfare is assessed on the basis of indicators for individual welfare aspects. Different research projects have worked on the development of animal welfare indicators. The Welfare Quality^®^ project [17] has set standards in this field of research and has since become a commonly used reference for animal welfare indicators. Due to the comprehensiveness of the assessment, which results in a high survey effort of six or more hours per farm [18], the application of the entire Welfare Quality^®^ protocol is unsuitable for use in the design of support measures. As a consequence, an approach which concentrates on the most important animal welfare issues was adopted for our indicator selection. The Welfare Quality^®^ protocol for dairy cows does play an important role in this approach, but not all indicators are included, and additional indicators were selected to address specific problems of the German dairy sector.

### 1.2. Determination of Threshold Values and Measure Design

In addition to suitable indicators, threshold values are essential components of results-oriented policy measures. The threshold value is the indicator value up to which a farm can receive a payment and above which an animal welfare payment is not considered acceptable. For the design of an animal welfare measure, the definition of such threshold values is necessary for each of the selected indicators. Their definition is based on value concepts, and it can therefore not be achieved solely on the basis of scientific knowledge. Science can nevertheless contribute by testing approaches for the determination of such values. In principle, normative and status quo-based procedures can be used to define threshold values. In normative approaches, a value is determined in a political or societal debate. This has the advantage that societal goals or a situation desirable from the point of view of animal welfare can be defined, regardless of the actual setting. Status quo-based methods are oriented towards the current situation. They have the advantage of “not bypassing reality” as well as avoiding complex value discussions that are difficult to resolve in consensus. Risks associated with the status quo-based approach are (A) that if prevalences are very high in practice (e.g., lameness), a problematic situation is rendered acceptable and (B) that in the case of very low prevalences, a minor deviation on a farm leads to the assessment of a situation as “problematic” that might be acceptable with respect to animal welfare.

## 2. Materials and Methods

### 2.1. Identification of Suitable Indicators

#### 2.1.1. Selection of Indicators by Experts

The initial identification of suitable indicators was carried out in a two-stage process involving scientists and practitioners. First, an indicator-database was compiled based on a comprehensive literature review. We considered international publications which focus on on-farm animal welfare assessments, which refer to production systems in dairy cattle similar to those in Germany, e.g., the Welfare Quality^®^ assessment protocol for cattle [18] and the “EFSA-toolbox“ with animal-based indicators to assess animal welfare of dairy cows [19]. Furthermore, we took scientific studies into account which address methodological issues of welfare assessments, such as [20,21,22,23,24,25], and various welfare indicators described by the different authors of the “Welfare Quality Reports No. 11” [26].

This “database” was used to assemble a list of 82 indicators. These were presented in a written survey to 42 farm animal welfare scientists from German-speaking countries (Germany, Austria and Switzerland) to compile a list of indicators identified as suitable according to scientific criteria of validity and reliability. In addition to scientific criteria, the suitability for a problem-oriented approach (Do the indicators address the most important problem areas in dairy farming in Germany?) was the main selection criterion. The contacted researchers work on relevant topics of farm animal husbandry in universities or other research institutes and have expertise in welfare issues of dairy cows. We chose these three countries for several reasons: (I) We assume that (due to the lively scientific exchange between the German-speaking countries and the geographic vicinity) researchers from these countries are informed about the circumstances of dairy farming, typical productions diseases and specific animal welfare problems in Germany. (II) Germany, Austria and Switzerland implement similar agricultural support measures (i.e., pasture premium, premium for straw bedding) which helped the scientists to understand the research question. (III) Due to the common language, we were able to involve a larger community (than just German scientists) without having to translate the survey documents. Most of the 42 scientists approached were from Germany, 5 were based in Austria 6 in Switzerland.

To reduce heterogeneity, the survey was conducted as a two stage Delphi study. This is a systematic, multi-stage survey procedure with a feedback of the aggregated anonymised results to the participants. A frequently pursued goal of Delphi surveys is to determine and qualify the views of a group of experts on a diffuse issue [27]. In the context of animal welfare and livestock production, Delphi surveys are a frequently used method [28,29,30,31,32].

The initial response rate was 50%, leading to 21 responses in the first round, with an even distribution across gender and origin (15 replies came from Germany, 2 from Austria and 3 from Switzerland, respectively). In the second round, 17 scientists used the opportunity to adjust their information.

To include the experiences of practitioners in the selection of indicators, representatives of agricultural interest groups and animal welfare NGOs and those of inspection bodies for organic farming and RDP measures, along with agricultural consultants, were invited to a group discussion (“practitioner workshop”). Group discussions are a reliable instrument “not to infer but to understand, not to generalize but to determine the range” [33] and have proven valuable in the selection of indicators [34]. The 20 participants had the task of evaluating the indicators selected by the scientists with regard to their practicability and suitability for measuring animal welfare in the context of an animal welfare support measure. Again, the relevance of the indicators with respect to the most important animal welfare problems (problem-oriented approach) was emphasised. The participants were informed about the indicators selected by the scientists in the Delphi study and had the opportunity for discussion. They were then asked to rank the indicators presented. For this purpose, small groups were formed and the participants were provided with stickers indicating agreement or disagreement, which they placed on posters with the pre-selected indicators. In a written follow-up, the participants were also asked to define threshold values for the selected indicators.

#### 2.1.2. On-Farm Testing of the Project Indicators

The indicators, which were approved by at least two-thirds of the participants of one of the two groups (scientists and practitioners) and not less than half of the second group, were included in a list of eleven “project indicators”. These project indicators were subsequently tested in an on-farm survey comprising 115 dairy farms to assess their practicability. Additionally, some indicators which were mentioned in the discussion with the practitioners such as “broken tails” and “Percentage of cows with milk fat-protein-ratio < 1.0 as an indication of rumen fermentation disorders” were also tested. The on-farm survey was also used to provide the database for the validation of the project indicators with the Welfare Quality^®^ assessment tool. To this end, all indicators of the Welfare Quality^®^ assessment protocol for cattle [18] were also surveyed. The WQ^®^ protocol [18] follows a “bottom-up” approach. In a first step, around 30 animal-based indicators are collected. These indicators are aggregated into twelve animal welfare criteria, which are then compressed to provide for an assessment for four animal welfare principles. In a fourth step, an “overall welfare score” is calculated and classified into four categories (“excellent”, “enhanced”, “acceptable” and “not classified”). At the level of animal welfare principles and criteria, a value of 100 corresponds to the best and a value of 0 to the worst result, while a value of 50 describes a “neutral” situation. Values from 0 to 20 are considered as “unacceptable” (“not classified”), an improvement is also required for values between 20 and 50 (“acceptable”) and should be improved to values between 50 and 80 (“enhanced”), whereas values between 80 and 100 (“excellent”) represented a very good situation [35]. In order to ensure good inter-observer-reliability, a training course was held for the four-member survey team, with inter-observer reliability tests [36] showing sufficient to very good agreement between the project staff.

The 115 farms participating in the survey were selected using a stratified random sample from farms participating in an action-oriented animal welfare measure (*n* = 3600 farms) and support to organic farming in the federal states of North-Rhine, Westphalia (*n =* 62) and Mecklenburg, Western Pomerania (*n* = 53) in Germany. The stratification made sure that a balanced number of farms were included in the survey with respect to the representation of the two federal states, organic and conventional farming and the sub-measures of the support (A: summer grazing, *n* = 27; B: loose housing on straw, *n* = 32 and a combination of A and B + 3 farms receiving support for organic farming, *n* = 56). The actual sampling within the defined subgroups was carried out as a random selection. The status quo of animal welfare was recorded for the 115 dairy farms (46 organic and 69 conventional) between November 2013 and May 2014. All surveyed farms had loose housing systems (75 with cubicle housing and 40 with free, deeply bedded lying areas), the mean herd size was 155 dairy cows and the annual milk yield per cow was 8137 kg on average. Table 1 presents the key data of the project farms.

#### 2.1.3. Statistics

In the two stage Delphi study, the values stated by the experts in the first round of the survey were evaluated descriptively and reported back to all participants, anonymised and aggregated as descriptive statistical parameters (mean, minimum, maximum, median, number of answers). 

For the analysis of the on-farm survey, individual animal-related data were converted into prevalences on herd level. The data evaluation was carried out with the program SAS^®^ 9.4 (SAS Institute Inc., Cary, NC, USA).

### 2.2. Determination of Threshold Values and Measure Design

Within the framework of the project, normative threshold values were collected from the scientists involved in the indicator selection as well as from the practitioners who participated in the group discussion (“practitioner workshop”). These values were compared with the indicator results on the surveyed farms, and the findings used to derive recommendations for an appropriate procedure for the definition of threshold values.

For the final design of the animal welfare measure, another group discussion, this time involving seven representatives from extension services and from agricultural ministries was carried out (“expert workshop”). The subjects discussed included the remuneration model of the measure (including the proposed thresholds) as well as additional requirements needed to address all dimensions of animal welfare.

## 3. Results

### 3.1. Identification of Suitable Indicators

#### 3.1.1. Selection of Indicators by Experts

As a result of the Delphi survey and the practitioner workshop, a list of eleven indicators (Table 2) was identified as suitable for a results-oriented animal welfare measure for dairy cows.

These indicators were subsequently tested in the on-farm survey. Based on the results of the analysis, the following indicators were excluded from the final list (a more detailed explanation is given in Section 4):

- Lameness: Percentage of severe lameness (2), due to collinearity with the indicator “prevalence of clinical lameness”;
- Lying behaviour/Cow Comfort Index: Percentage of cows in stalls that are lying down (7), because of difficulties in on-farm data collection;
- Calf mortality: Percentage of euthanized and deceased calves (11), as reliable data proved to be unavailable.

In return, two additional indicators were added to the list:

- Percentage of cows with broken tails, an indicator suggested by practitioners, which proved to be relevant;
- Percentage of cows with milk fat-protein-ratio < 1.0 as an indication of rumen fermentation disorders, which also occurred frequently on the surveyed farms and for which data is readily available.

#### 3.1.2. Testing of the Project Indicators

##### Results of Selected Indicators

The indicator values from the 115 project farms are shown in Table 3. Some of the indicators, such as the prevalence of dirty cows, are characterised by a wide range, i.e., the results of the individual farms are generally relatively far apart. Other indicator values were close to each other for most farms, but a few farms have extreme values. This was true for the prevalence of cows with severe swellings or lesions on carpus or tarsus and for the indicator cows with broken tails.

##### Results of Welfare Quality^®^ Assessment

**Figure 2:**
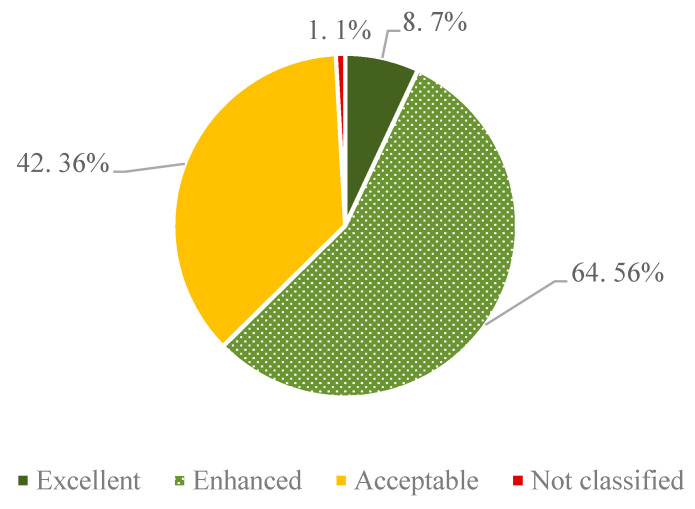
Results of the “overall welfare score”, according to the Welfare Quality^®^ assessment protocol, in the 115 project farms.

In the “overall welfare score” according to the Welfare Quality^®^ protocol [18], eight farms (7%) were classified as “excellent”, 64 farms (56%) as “enhanced”, 42 farms (36%) as “acceptable” and one farm as “not classified” (Figure 2). For results of the twelve animal welfare criteria as well as the aggregation into four animal welfare principles, see Table A1 in Appendix B.

### 3.2. Determination of Threshold Values and Measure Design

#### 3.2.1. Defining Threshold Values for the Animal Welfare Indicators

**Figure 3:**
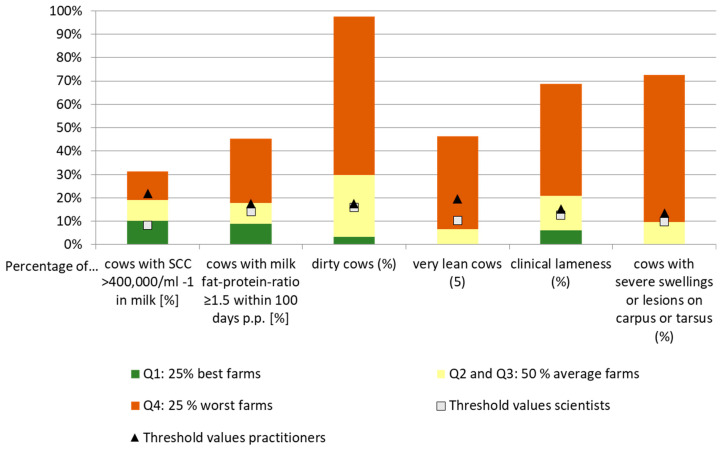
Normative versus status quo-based threshold values: A comparison of the normative values of scientists (*n =* 12–17) and practitioners (*n =* 8–9) with the results of the on-farm survey (*n* = 115).

The on-farm survey provided indicator values for 115 farms. In Figure 3, the normative threshold values of scientists and practitioners are contrasted with the on-farm situation. For the sake of clarity, not all project indicators, but a selection of six indicators, is presented in a bar chart. The values of those indicators not depicted can be found in Appendix B, Table A2.

The indicator results are structured into quartiles; the first quartile (Q1) represents the best 25%, the second and third quartile (Q2, Q3) are aggregated into the group of the “50% average farms” and the fourth quartile (Q4) represents the worst 25% farms (see Table 3 and Table A2 in Appendix B).

1. Mastitis: Percentage of cows with SCC > 400,000 mL^−1^ in milk: The 25% best farms (Q1, green bar) had 0–10.1%, the 25% worst farms (Q4, red bar) had 19–31.4% cows with SCC > 400,000 mL^−1^. The scientists set their threshold values at 8% (in the green bar), practitioners at 22% (in the red bar).
2. Ketosis: Percentage of cows with milk fat-protein-ratio ≥1.5 within 100 days p.p. ranged from 0–8.6% in Q1 and from 17.4–45.0% in Q4. Here the scientists’ thresholds were 14% and the practitioners’ thresholds 18%, both located in the yellow bar.
3. Cleanliness: Percentage of dirty cows: The 25% best farms had 0–3.3% dirty cows, the 25% worst farms had 29.7–97.5%. Again, the thresholds were located in the yellow bar and amounted to 16% (scientists) and 17% (practitioners).
4. Body condition: Percentage of very lean cows: The 25% best farms had 0%, and the 25% worst farms had 6.7–46.3% very lean cows. This is the only indicator where the two thresholds were located in the red bar (10% scientists; 20% practitioners).
5. Lameness: Prevalence of clinical lameness: The 25% best farms had 0–6.1%, the 25% worst farms (red bar) had 20.8–68.8% lame cows. Again, the thresholds were located in the yellow bar (13% scientists, 15% practitioners).
6. Integument alterations on limbs: Percentage of cows with severe swellings or lesions on carpus or tarsus: the 25% best farms had 0% cows with integument alterations on limbs, the 25% worst farms (red bar) had 9.8–72.5%. The scientist’s thresholds were set at 10% and those of practitioners at 14% for this indicator and were in the yellow and red bar, respectively.

Generally, the threshold values of the practitioners are higher than those of the scientists for all indicators (also the ones not depicted in the Figure 3 (see Table A2 in Appendix B). For most of the indicators—if the normative thresholds were applied—farms having average indicator results (yellow bar, 50% average farms) would be eligible for support in an animal welfare measure.

#### 3.2.2. Remuneration Model

The remuneration model specifies which farms would be entitled to receive payments from a results-oriented animal welfare measure. As the normative approach to the setting of threshold values proved to be unsuitable, status-quo threshold values were used for the development of a remuneration model, which is an adapted version of Spoolder’s assessment method [29]. This approach was discussed and agreed upon in the “expert workshop”.

In the remuneration model, for each individual indicator, a farm whose indicator value corresponds to that …

- achieved by the 25% best farms, indicating “good animal welfare”, would receive a payment (per cow and year) in a results-oriented animal welfare measure;
- of the 50% average farms indicating “acceptable animal welfare”, would not receive a payment for this indicator;
- of the 25% worst farms, “unacceptable animal welfare”, would lead to exclusion from the animal welfare measure.

In the expert workshop, the question was raised of whether one indicator in the category of “unacceptable animal welfare” (Q4) should be permissible. The participants agreed that this option would generally be conceivable (i.e., for indicators such as cleanliness) but not for indicators which are of particularly high relevance for animal welfare due to their painfulness for the animal. This criterion should be applied to the indicators “Lameness: Prevalence of clinical lameness” and “Mastitis: Percentage of cows with SCC > 400,000 mL^−1^ in milk (%)”.

##### Comparison of Results of Project Indicators and Welfare Quality^®^ Assessment

The comparison of the project indicators (operationalised in the remuneration model) with the Welfare Quality^®^ protocol showed a limited degree of consistency between the two systems. Farms receiving a poor overall rating in Welfare Quality^®^ also scored poorly based on the set of indicators selected in the project (Table 4). On the other hand, a large number of farms which were classified as “enhanced” in Welfare Quality^®^ (43 farms) would not have been eligible for an animal welfare measure in the assessment based on the project indicators. Furthermore, an important number (31) of farms, which only received the classification “acceptable” in the Welfare Quality^®^ assessment, would have been eligible for support in the assessment based on the project indicators.

## 4. Discussion

### 4.1. Identification of Suitable Indicators

The indicator selection was based on a literature review, a written Delphi survey with scientists, a group discussion with stakeholders (“practitioner workshop”) and the on-farm trial of the pre-selected indicators. The quality of the results of Delphi surveys depends crucially on the response rate, the selection of the experts involved and their qualifications [38]. Group discussions, on the other hand, can only provide robust results if all relevant stakeholder groups are involved. If, for example, animal welfare NGOs, or the extension service, were not invited to participate in the selection of suitable animal welfare indicators, the results of such a discussion would be questionable.

The response rate of the Delphi study was 50% in the first round and 80% in the second round. This is a common response rate for expert surveys (see, for example, [39]). In the basic literature describing the procedures of Delphi surveys, [40] response rates of 30% in the first round and between 70–75% for the following round(s) are considered satisfactory. As the contacted researchers were selected because of their expertise in the on farm animal welfare of dairy cows, we expect the quality of results of the Delphi study to be robust. This applies also to the group discussions with the practitioners, as all relevant stakeholder-groups were invited and represented.

Scientists and practitioners were broadly in agreement (see Table 2) and, for a majority of indicators, the on-farm trial demonstrated practicability. However, three of the initially selected indicators were excluded from the final list:

1. “Lameness: Prevalence of severe lameness” because of multicollinearity with “Lameness: Prevalence of clinical lameness”.
2. “Lying behaviour/Cow Comfort Index: proportion of cows in stalls that are lying down”. The Cow Comfort Index is an indicator of lying behaviour. It was primarily designed for cubicle housed cows [22] and is not suitable for assessment of other housing systems (e.g., deep litter), which are also common in dairy farming in Germany (mostly in organic farms). Furthermore, our results show large farm-specific differences (range 10.6–100%), which partly resulted from the difficulties of finding a suitable time window.
3. “Calf mortality: Percentage of euthanized and deceased calves”, because only inconsistent data was available, resulting in a systematic underestimation of calf mortalities. The cattle register data, which allows for a reliable calculation of mortality of adult cattle, is unsuitable for the calculation of calf mortality as data is not reliably recorded in the first week of the calf’s life. This is due to the fact that entry into the system is only mandatory from the eighth day of life onwards, leading to a situation where some farms record calves that die in the first week while other farms do not.

Indicators were not only excluded from the list, some were also added to the list. In the “practitioner workshop”, the participants advised to investigate in the on-farm survey if broken tails occur and to include this as a new indicator to the list, if this should be the case. As broken tails have been ascertained on 5.6% of the cows on the 115 surveyed farms with one fifth of farms exceeding 10% of cows with broken tails, and it is easy to assess, this indicator was added to the list. Broken tails as welfare-indicator is also included in other indicator sets [41], because tail injuries or broken tails are extremely painful for the affected cow; high prevalence can be caused by mechanical injuries from slurry-scrapers and brushes as well as rough cow handling by farm staff [41]. Furthermore, the indicator “Percentage of cows with milk fat-protein-ratio < 1.0” has been suggested by the practitioners and was consequently included as it can indicate rumen fermentation disorders [42]. These are often a result of a very starchy diet which is unsuitable for ruminants as it can be a risk factor for rumenitis or subclinical ruminal acidosis and subsequently also laminitis. This animal welfare problem was highlighted in the on-farm survey (8.9% of the cows had a milk fat-protein-ratio < 1.0), and the indicator can be generated from existing data without additional effort.

The selected indicators show a high degree of concordance with other studies/projects which focussed on animal-based indicators. For example, nine of the above-mentioned project indicators are also included in the list of 15 indicators recommended for on-farm self-monitoring [43,44] and eight of the project indicators can be found in the “AssureWel”-list of eleven indicators for organic farming control of the “Soil Association” [41].

The differences in the animal welfare assessment of the Welfare Quality^®^ protocol with the assessment based on the project indicators had several reasons. Here we focus on the explanations as to why farms scored well on project indicators but performed poorly in the Welfare Quality^®^ assessment, as this could lead to a situation where farms with poor animal welfare would receive premiums under an animal welfare support measure. The reasons why farms that received a good rating based on the project indicators were classified as only “acceptable” in the Welfare Quality^®^ assessment were deficiencies in water supply (principle “Good Feeding”), pain induced by management procedures (disbudding) and weak points in the principle “Appropriate behaviour“, mainly influenced by a resource/management-based impact: access to pasture [45]. These aspects are not part of the list of project indicators.

The selected indicator list does not cover all dimensions of animal welfare [15] as it lacks indicators to assess animal behaviour. The ability to carry out normal behaviour possibly was not seen as an important animal welfare problem of dairy cows by the scientists and practitioners involved in the selection process. Possibly the fact that the assessment of animal behaviour using animal-based indicators requires a considerable amount of time also played a role in the selection decisions. Emotional state and water supply are also not part of the list, because no suitable animal-based indicators (which are a precondition for results-oriented measures) exist [18]. We address this issue, not by introducing changes to the set of indicators selected by scientists and practitioners, but through measure design (see Section 4.2).

Nearly all farms included in the study participated in the action-oriented animal welfare support measure (M14). A high number of organic farms are among these supported farms. This selection was based on the consideration that farms receiving support in such a measure, as well as organic farms, would probably be willing to participate in a future results-oriented animal welfare measure.

It is likely that the conventional farms participating in the animal welfare support measure have above average results with respect to the surveyed animal welfare indicators. For organic farming, only a few reviews are available which compare and evaluate the animal welfare situation with that on conventional farms. The current analysis found no fundamental differences in the animal welfare situation of the two farming systems, apart from parasitic diseases [46,47,48], udder health and antibiotic resistance [49].

In a general comparison of indicator results of the project farms (Table 3 and Figure 2) with the literature, the project farms achieved better results for the “overall welfare score” compared to Kirchner et al. [50] and Gratzer et al. [51]. This is due to higher scores for the principles “Good Housing”, “Appropriate Behaviour” and “Good Health”. In contrast to other Welfare Quality^®^-assessments [52,53,54,55,56], in which no farm was rated “excellent”, this was the case in 8 out of 115 farms (7 organic and 1 conventional). This finding can be explained by the fact that most of the project farms participated in an action-oriented animal welfare policy measure. Many of the farms in this support measure also managed their holdings according to organic guidelines, resulting in a higher proportion of organic farms in our sample compared to other studies. Compared to the results presented by Heath et al., for 92 farms in England and Wales, the dairy farms in our study had better results, especially with respect to the WQ^®^ principle “Appropriate Behaviour”, which could be explained by the higher share of organic farms and the respective requirements (e.g., pasture).

Overall, our results of the WQ^®^-principles and criteria (see Table A1 in Appendix B) were comparable to Schulz et al., who assessed the WQ^®^-protocol in 34 farms in Germany (19 organic, 15 conventional farms) and also reported benefits in terms of a better welfare in organic dairy farms compared to conventional farms [57].

The indicator results generated on the 115 project farms should nevertheless not be regarded as representative for all German dairy farms. As the main task of the on-farm indicator survey was not to generate valid data on the animal welfare situation of dairy cows, but to test the feasibility of the selected indicators, the question of representation does not play an important role in the framework of this study.

### 4.2. Determination of Threshold Values and Measure Design

A comparison of the threshold values defined normatively by scientists and practitioners with the values recorded in the on-farm survey showed that the normative approach is associated with considerable difficulties (see Figure 2). On the one hand, value differences between the two social groups (science and practice) become obvious. On the other hand, the application of the normative threshold values can lead to a situation where farms would receive an animal welfare payment even when they belong to the group of the 25% worst farms with respect to one or several animal welfare indicator/s. For most of the indicators—if the normative thresholds were applied—farms having average indicator results (yellow bar) would be eligible for support in an animal welfare measure. This would lead to a measure that would reward average animal welfare—a situation which cannot be considered an efficient use of public funds.

Therefore, when setting threshold values for animal welfare indicators, the status quo on the farms should be considered. As no representative data is available for most relevant animal welfare indicators, values from scientific studies with limited sample sizes have to be used until a better database is available (e.g., with respect to lameness, an overview can be found at https://www.cattle-lameness.org.uk/research, accessed on 19 January 2021). The use of threshold values derived from the status quo should not replace the discussion about the socially desired level of animal welfare in livestock farming, but ensure that support payments for an animal welfare measure are not disbursed to farms whose animal welfare situation is only average or even in the lower quartile. This combination of status quo and normative approaches has proven successful in projects which provide reference values for the animal welfare self-assessment according to the Animal Welfare Act [58] in Germany [59,60].

Because the indicators selected in the project cover only aspects of animal health, an animal welfare support measure should contain action-oriented requirements in order to be able to consider the dimensions “behaviour” and “emotions” of animal welfare. In the “expert workshop”, the following action-oriented requirements were identified to be included in the animal welfare measure to enable the cows to carry out normal behaviour and ensure access to water:

- cow to cubicle ratio of max. 1:1;
- sufficient number of functional drinkers;
- animal to feeding place ratio of max. 1:1; access to pasture for all cows.

In order to include aspects of “emotional state”, the support measure requirements should also prescribe the use of anaesthesia, sedatives and analgesia when disbudding.

With respect to the payments for the participation in a possible future results-oriented measure, this should consist of two components: a base premium for compliance with the action-oriented requirements and payments for each indicator where the farm has achieved the required result.

The remuneration model defined in the project with the ’25-50-25 split’, discussed and agreed upon in the “expert workshop”, may seem arbitrary, and of course other models (such as 33-33-33 or even 50-25-25) are conceivable as well. It should be seen as one possible approach to address the challenge of a results-oriented support measure, not as the only solution.

With respect to the payment to the farm, the amount per cow would not necessarily have to change from the one disbursed in the current system (50-130 Euro per cow per year, see Appendix A), but generally the “right” amount is often determined in a “trial and error phase” at the beginning of the implementation of a new measure (if too many farms apply for participation in the measure, the amount would be reduced, and vice-versa).

Even though the empirical research for our analysis was carried out in two federal states of Germany and thus has very limited geographical coverage, the results are relevant in the EU-context. As animal health—an important dimension of animal welfare—is influenced far more by management than by requirements on the housing system, the large number of the action-oriented animal welfare measures implemented in the EU fail to achieve animal welfare. With the inclusion of results-oriented elements, the performance of these measures could be increased substantially.

## 5. Conclusions

With a combination of action-oriented requirements and results-oriented indicators, all dimensions of animal welfare: health, behaviour and emotions (e.g., by avoiding fear and pain when disbudding) can be covered in support measures for dairy cows. Due to the higher complexity of such support compared to purely action-oriented measures, a scientifically accompanied trial phase with a limited number of farms is recommended.

With regard to the role that support measures can play in improving animal welfare in livestock farming, it should be noted that voluntary support measures are not suitable for preventing violations of animal welfare laws. They are also not adequate for the improvement of the situation on farms which have severe animal welfare problems, as these measures will be taken up primarily by farms that are interested in animal welfare and consequently, on average, achieve a relatively good level of animal welfare. To improve the situation on farms with relevant animal welfare problems, other approaches, such as a tightening of animal welfare legislation, increased controls and more effective enforcement of animal welfare legislation, would be appropriate [61].

## Figures and Tables

**Table 1:** Selected key data of the 115 project farms in 2014, mean values and range (min–max) at herd level.

Item	Unit	Mean (min–max)
Herd size	dairy cows	155 (21–1495)
Milk yield ^1^	kg/cow/year	8137 (4405–11,988)
Herd age ^2^	years	4.9 (3.5–6.9)
Culling rate ^3^	%	27.5 (8.7–56.7)
Mean productive life time ^3^	years	3.3 (1.9–7.9)
Housing system: 75 farms with cubicle housing and 40 farms with free, deeply bedded lying areas		

^1^ Annual moving average milk yield from milk recording data 2014 (*n* = 107). ^2^ Calculations are based on monthly milk recording data, 2014 (*n* = 106). ^3^ Based on milk recording data, calculation is carried out with the module “Betriebsvergleich” (farm comparison) of ITB-Controlling software from dsp-Agrosoft GmbH, Pareetz (*n* culling rate = 103; *n* productive life time = 105).

**Table 2:** Scientists and practitioners indicator selection.

Scientists (*n* = 21/17) ^1^	Practitioners (*n* = 20)	Indicator
✓	✓	Mastitis: Percentage of cows with SCC > 400,000/mL in milk (%)
✓	✓	Ketosis: Percentage of cows with milk fat-protein-ratio ≥1.5 within 100 days p.p. (%)
✓	✓	Cleanliness: Percentage of dirty cows (%)
✓	✓	Body condition: Percentage of very lean cows (%)
✓	✓	Lameness: Percentage of clinically lame cows (%)
✓	✓	Lameness: Percentage of severely lame cows (%)
✓	✓	Integument alterations on limbs: Percentage of cows with severe swellings or lesions on carpus or tarsus (%)
✓	✓	Integument alterations, other body regions: Percentage of cows with severe swellings or lesions on other body regions (%)
☑	✓	Lying behaviour/Cow Comfort Index: proportion of cows in stalls that are lying down
✓	☑	Cow mortality: Percentage of euthanized and deceased cows (%)
✓	☑	Calf mortality: Percentage of euthanized and deceased calves (%)

✓ Indicators with ≥ 66% acceptance. ☑ Indicators with <66% but >50% acceptance. ^1^ Number of responses in Delphi survey in first and second round, respectively.

**Table 3:** Indicator values of the 115 farms surveyed in 2014 (mean, median = MED, minimum = min, maximum = max and quartiles = Q). Indicators excluded from the final list have a red font colour. Indicators added to the final list have a green font colour.

Indicator		Mean	Min	Q1	MED	Q3	Max	*n*
Mastitis: Cows with SCC > 400,000/mL in milk ^1^	%	14.9	2.6	10.1	13.3	19.0	31.4	106
Ketosis: Cows with milk fat-protein-ratio ≥1.5 within 100 days p.p.^1^	%	14.5	0.3	8.6	12.1	17.4	45	106
Rumen fermentation disorders: Cows with milk fat-protein-ratio < 1.0 ^1^	%	8.9	0.7	4.0	6.9	10.8	44.1	106
Cleanliness: Dirty cows ^2^	%	20.1	0.0	3.3	12.5	29.7	97.5	115
Body condition: Very lean cows ^2^	%	4.7	0.0	0.0	3.1	6.7	46.3	115
Lameness: Clinically lame cows ^2^	%	14.7	0.0	6.1	12.1	20.8	68.8	115
Lameness: Severely lame cows ^2^	%	1.7	0.0	0.0	0.0	2.7	12.5	115
Integument alterations on limbs: Cows with severe swellings or lesions on carpus or tarsus ^2^	%	7.9	0.0	0.0	3.0	9.8	72.5	115
Integument alterations, other body regions: Cows with severe swellings or lesions on other body regions ^2^	%	10.8	0.0	2.5	8.0	12.5	56.3	115
Cows with broken tails ^2^	%	5.6	0.0	0.0	2.4	6.3	48.8	115
Lying behaviour/Cow Comfort Index ^3^: Cows in stalls that are lying down	%	79.5	10.6	75.9	81.9	87.3	100	115
Cow mortality: Euthanized and deceased cows ^4^	%	2.8	0.0	1.1	2.4	3.6	31.3	105
Calf mortality: Euthanized and deceased calves ^5^	%	7.9	0.0	2.4	5.1	11.7	31.3	105

^1^ Calculations were based on monthly milk recording data (*n* = 106). ^2^ Calculations of prevalences on farm-level were based on individual animal assessments during the farm visits in winter 2013/14. ^3^ Cow Comfort Index = number of cows observed lying in stalls/lying area divided by the total number either lying or standing in a stall/with at least two limbs on the lying area; modified according to [22]. ^4^ The mortality rates are calculated as the average of the past three calendar years (2012–2014) based on the “HIT”-data (cattle register data in Germany) [37] (*n* = 105). ^5^ See 4. Calf mortality was calculated from the 8th day of life, as the data entries for the first week of life in the HIT-database are not reliable due to the documentation requirements. [37] (*n* = 105).

**Table 4:** Welfare Quality^®^ assessment in comparison to the qualification of farms to participate in a results-oriented support measure.

Item	Project Indicator Assessment (Remuneration Model)		
Welfare Quality^®^ Overall Assessment	Eligible (max 1 Indicator in the Lower Quartile)	Not Eligible (>1 Indicator in the Lower Quartile)	Total
Excellent	6/16%	2/3%	8/7%
Enhanced	21/55%	43/56%	64/56%
Acceptable	11/29%	31/40%	42/37%
Not classified	0/0%	1/1%	1/1%

## Data Availability

None of the data were deposited in an official repository. The data that support the study findings are available upon request.

## Appendix A

The large increase in LU in 2019 is due to the introduction of M14 in Croatia as well as very high numbers in Italy and Romania. It is generally difficult to validate the data, and the most recent year especially (2019) should be treated with caution as it might still be subject to correction.

### Characteristics of the RDP Animal Welfare Support Measure (M14)

The most important requirements of Measure M14 “loose housing on straw” for dairy cows in North-Rhine, Westphalia (as an example for a typical action-oriented support measure) are: tie-stalls are not eligible for support:

- usable area of 5.5 m^2^ per animal;
- lying area with a straw-bed on solid floor on which all animals can lie down at the same time;
- animal to feeding space ratio of 1:1 or 1.2:1 in the case of continuous feeding.

For the farms participating in this measure, the payment is 80 Euro per cow per year.

For the measure “summer grazing”, the animals must have daily access to pasture from 16th of May to 15th of October, and the available grazing area must be at least 0.2 ha per LU.

The amount of the subsidy is 50 euros per LU per year or 40 euros for organic farms.

The two measures can be combined if all requirements are met, resulting in a payment of 130 Euro per cow.

Source: Guidelines for the promotion of husbandry methods on straw and summer grazing RdErl. d. Ministeriums für Klimaschutz, Umwelt, Landwirtschaft, Natur- und Verbraucherschutz—II A 4-62.71.10 v. 27.3.2015.

## Appendix B

**Table A1:** Overall assessment of the results of Welfare Quality^®^ assessment in 115 project farms presented as WQ^®^ principles and criteria in mean values, range (min-max).

WQ^®^ Principles and Criteria	All Farms (*n* = 115)
Good Feeding	49.0 (4.2–100)
1. Absence of prolonged hunger	74.5 (13.1–100)
2. Absence of prolonged thirst	51.7 (3.0–100)
Good Housing	66.7 (37.0–100)
3. Comfort around resting	47.2 (0.0–100)
4. Thermal comfort	100 (100–100)
5. Ease of movement	100 (100–100)
Good Health	49.3 (30.0–78.8)
6. Absence of injuries	62.4 (21.3–97.2)
7. Absence of disease	51.3 (30.2–86.0)
8. Absence of pain induced by management procedures	63.1 (20.0–100)
Appropriate Behaviour	57.7 (17.0–90.8)
9. Expression of social behaviour	83.4 (21.5–100)
10. Expression of other behaviour	51.8 (0.0–100)
11. Good human-animal relationship	60.8 (27.4–95.4)
12. Positive emotional state	84.7 (0.7–100)

**Table A2:** Normative threshold values (mean) given by 21 and 17 scientists in the two rounds of the Delphi survey (*n =* 12–17 with naming of threshold values for the single indicators) and 20 practitioners (*n =* 8–9 with naming threshold values) and their comparison with the results of the on-farm survey (*n* = 115, Quartile 1 and 3; Q = Quartile). Indicators excluded from the final list are green formatted. Indicators added to the final list are green formatted.

Indicator		Scientists	Practitioners	Farm Survey (*n* = 115)		
		with Naming of Threshold Values				
		*n* = 12–17	*n* = 8/9	Q1	Q3	*n*
Mastitis: Cows with SCC >400,000/mL in milk ^1^	%	8.4	21.8	10.1	19.0	106
Ketosis: Cows with milk fat-protein-ratio ≥1.5 within 100 days p.p.^1^	%	14.1	17.6	8.6	17.4	106
Rumen fermentation disorders: Cows with milk fat-protein-ratio <1.0 ^1^	%	*	*	4.0	10.8	106
Cleanliness: Dirty cows ^2^	%	16.0	17.4	3.3	29.7	115
Body condition: Very lean cows ^2^	%	10.2	19.6	0.0	6.7	115
Lameness: Clinically lame cows ^2^	%	12.6	15.3	6.1	20.8	115
Lameness: Severely lame cows ^2^	%	3.8	*	0.0	2.7	115
Integument alterations on limbs: Cows with severe swellings or lesions on carpus or tarsus ^2^	%	9.8	13.5	0.0	9.8	115
Integument alterations, other body regions: Cows with severe swellings or lesions on other body regions ^2^	%	9.5	13.8	2.5	12.5	115
Cows with broken tails ^2^	%	3.4	*	0.0	6.3	115
Lying behaviour/Cow Comfort Index ^3^: Cows in stalls that are lying down	%	74.6	76.1	75.9	87.3	115
Cow mortality: Euthanized and deceased cows ^4^	%	3.6	5.6	1.1	3.6	105
Calf mortality: Euthanized and deceased calves ^5^	%	6.2	*	2.4	11.7	105

^1^ Calculations were based on monthly milk recording data (*n* = 106). ^2^ Calculations of prevalences on farm-level were based on individual animal assessments during the farm visits in winter 2013/14. ^3^ Cow Comfort Index = number of cows observed lying in stalls/lying area divided by the total number either lying or standing in a stall/with at least two limbs on the lying area; modified according to [22]. ^4^ The mortality rates are calculated as the average of the past three calendar years (2012–2014) based on the “HIT”-data (cattle register data in Germany) according to Pannwitz [37] (*n* = 105). ^5^ See 4. Calf mortality was calculated from the 8th day of life, as the data entries for the first week of life in the HIT-database are not reliable due to the documentation requirements [37] (*n* = 105). * As the threshold query was made before the final indicator selection, there are some normative values missing.
